# Hepatic Abscess in a Returning Traveler with Crohn's Disease: Differentiating Amebic from Pyogenic Liver Abscess

**DOI:** 10.1155/2018/9593865

**Published:** 2018-05-29

**Authors:** Daria Gaut, Hannah Shull, Anthony Bejjani, Daniel Kahn

**Affiliations:** Department of Medicine, David Geffen School of Medicine, University of California Los Angeles, Los Angeles, CA, USA

## Abstract

Liver abscess is a rare but serious complication of Crohn's disease. Patients with Crohn's disease are at risk for pyogenic liver abscesses due to immunosuppressive therapy, fistulous disease, and intraabdominal abscesses. Inflammatory bowel disease patients are also known to have a greater prevalence of amebiasis compared to the rest of the population; however, a higher incidence of amebic liver abscess has not been reported. We describe a case of a liver abscess in a patient with Crohn's disease that was initially presumed pyogenic but later determined to be amebic in origin. Epidemiology, clinical presentation, diagnosis, and treatment of amebic and pyogenic liver abscesses are discussed.

## 1. Introduction

Pyogenic and amebic liver abscesses are the two most common types of hepatic abscesses. While both types of hepatic abscesses can present similarly, treatment differs significantly. Despite improvements in therapeutic modalities, pyogenic liver abscess remains a serious condition with a high morbidity and a mortality rate of up to 60% [1]. In contrast, amebic liver abscesses have a good prognosis if diagnosed and treated early. We report a case of liver abscess in a patient with Crohn's disease eventually diagnosed as an amebic abscess by serology that responded well to initial therapy.

## 2. Case Presentation

A 28-year-old male with Crohn's disease on azathioprine 100 mg daily in longstanding clinical remission presented to an outpatient gastroenterologist for sudden onset of right upper quadrant abdominal pain 8/10 in severity with radiation to his back. He also endorsed fevers but denied nausea, vomiting, any change in bowel movements, or rectal bleeding. Five months prior to admission, the patient had traveled to northern India and, while there, contracted “traveler's diarrhea,” which was self-limited and treated with loperamide.

At a gastroenterology clinic, a right upper quadrant ultrasound revealed a 46 mm hypoechoic lesion in the liver, correlating with the area of the patient's pain. The patient was then referred to the emergency room for further evaluation.

**Figure 1:**
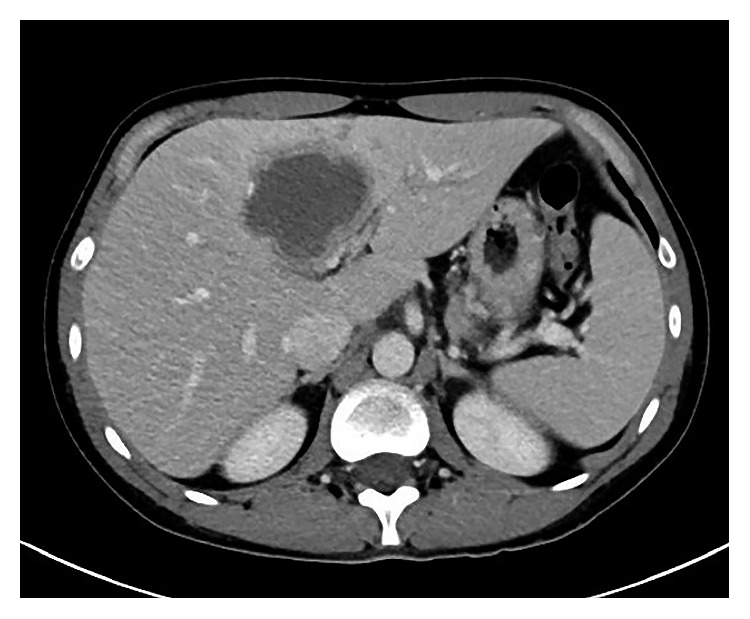
Abdominal CT scan on hospital admission revealing a complex lower attenuating 6.0 cm hepatic segment 4A/B lesion with a thick marginal rim, adjacent parenchymal inflammation, and biliary prominence.

In the emergency room, the patient was afebrile without hypotension or tachycardia. Labs revealed a white blood cell count of 9.91 × 10^3^/*μ*L, hemoglobin of 13.4 g/dL, platelets of 277 × 10^3^/*μ*L, aspartate aminotransferase of 18 U/L, alanine aminotransferase of 22 U/L, total bilirubin of 0.5 mg/dL, alkaline phosphatase of 64 U/L, and C-reactive protein (CRP) of 23.2 mg/dL. Computed tomography (CT) scan of the abdomen/pelvis showed a 6.0 cm × 5.0 cm low/intermediate density lesion in segment 4A/B of the liver with a thick marginal rim and adjacent parenchymal inflammation and biliary prominence (Figure 1). Due to concern for pyogenic abscess, the patient underwent drainage of the abscess by interventional radiology with 110 cc of reported “pus” removed “without anchovy appearance.” A drainage catheter was placed.

Following drainage, the patient was started on empiric ertapenem 1 g IV daily and metronidazole 500 mg PO TID. Azathioprine was held. Bacterial, fungal, and acid-fast cultures were negative from the drained fluid. Blood cultures were also negative, except one drawn 2 days into admission that resulted positive for *Staphylococcus epidermidis*, presumed to be a contaminant. Stool exam for ova and parasites resulted four days after admission with protozoa, later identified as *Entamoeba coli* trophozoites. The patient's second stool sample was positive for nonpathogenic protozoa, and the third stool sample was negative.

**Figure 2:**
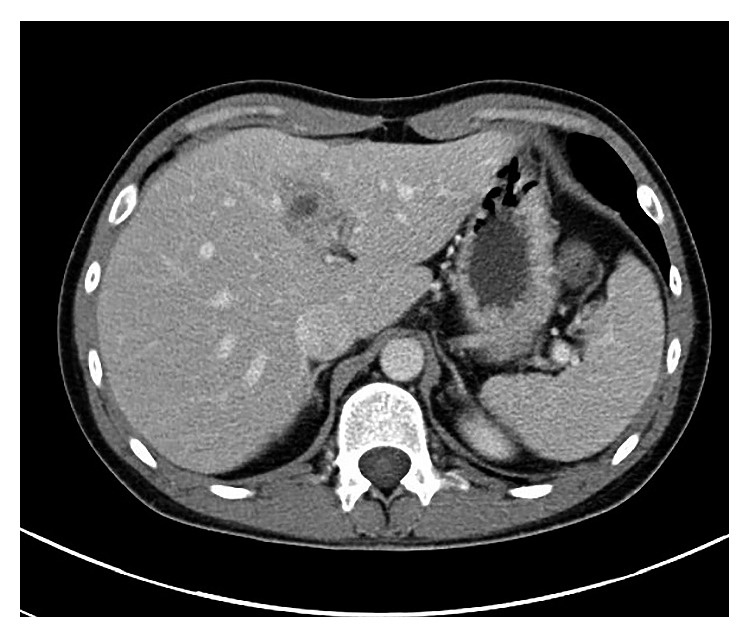
Abdominal CT scan 2 weeks after discharge demonstrating near total resolution of the previously seen hepatic segment 4 abscess without any drainable fluid collection.

The patient was discharged on ciprofloxacin 750 mg PO BID and metronidazole 500 mg PO TID for an anticipated three-week antibiotic course from the day of drainage. *Entamoeba histolytica* IgG antibody returned positive three days after discharge, supporting the diagnosis of amebic liver abscess. The *Entamoeba histolytica* stool antigen returned negative. *Echinococcus* and *Strongyloides* serologies also returned negative. The drain was removed by interventional radiology 1 week after discharge. Repeat CT scan of the abdomen/pelvis 2 weeks after discharge revealed near complete resolution of the abscess with no drainable fluid collection (Figure 2).

## 3. Discussion

Hepatic abscesses are defined as a suppurative collection within the liver parenchyma infected with either bacterial, parasitic, or less-commonly fungal organisms. Incidence varies according to geographic regions, with amebic being the most common in Southeast Asia and Africa and bacterial (or pyogenic abscess) being the most frequent in Western countries [2]. Amebic liver abscesses are also more common in younger adult males and tend to present with solitary right lobe abscesses [1]. Pyogenic liver abscesses, more common in adults over 50 years old, may result from portal vein pyemia (in the setting of intrabdominal infection), biliary tract disease, or hematogenous seeding and often present with multiple abscesses [1, 3].

Symptoms of pyogenic and amebic liver abscesses are similar and most commonly include fever and abdominal pain. Nausea, vomiting, malaise, and weight loss can also occasionally be seen [4]. Laboratory findings include leukocytosis, elevated CRP, and sometimes liver function test abnormalities [5]. Given that neither symptoms nor laboratory testing are specific, diagnosis relies largely on imaging. Both ultrasound and CT carry a sensitivity of 96–100% for detection of hepatic abscesses; however, they are not able to definitively differentiate the microbiological etiology [5].

Serology can be used to confirm the diagnosis of an amebic liver abscess. Most patients will not have detectable parasites in their stool, as a liver abscess frequently occurs without colitis, but serum antibodies are up to 100% sensitive [6] and 85–95% specific after one or more weeks of symptoms in patients with an amebic liver abscess [3]. As antibodies may persist after acute infection, serologic tests do not have the ability to distinguish past from current infections [3]. In a nonendemic setting, however, a positive serologic test in addition to an imaging finding of liver abscess is strongly suggestive of amebic etiology. Histopathologic samples of abscess fluid can also be used to confirm the diagnosis. Aspirated material from an amebic liver abscess contains acellular, proteinaceous debris with necrotic hepatocytes that form a brown fluid referred to as “anchovy paste” [7]. Trophozoites can also be seen in a minority (<20%) of aspirates [8]. This is in contrast to aspirates from pyogenic liver abscesses which consist of purulent material with bacteria and neutrophils that are readily apparent by Gram stain.

Treatment of pyogenic liver abscess usually involves both antibiotic therapy and adequate drainage, though there have been some studies to suggest that small abscesses 3–5 cm can be treated by antibiotics alone [9]. Amebic liver abscesses generally resolve with just medication. Indications for percutaneous drainage include if the abscess is large (>10 cm in diameter), subcapsular, high risk for rupture, superinfected, or if there is poor response to medical treatment [2].

Empiric antibiotic choice for a pyogenic abscess should be directed at the microbes typically responsible, which include aerobic enteric Gram-negative bacilli (*Escherichia coli* and *Klebsiella pneumoniae*, the latter particularly prevalent in Asia), Gram-positive cocci (most commonly the *Streptococcus milleri* group but also including *Staphylococcus aureus*, *Streptococcus pyogenes*, and *Enterococcus* spp.), and anaerobes (*Bacteriodes*, *Fusobacterium*, *Actinomyces*, and anaerobic streptococci) [10, 11]. Reasonable single-agent regimens include carbapenems or piperacillin-tazobactam, or a combination regimen of a third-generation cephalosporin plus metronidazole can be used [12]. The latter is preferred in most cases in which drug-resistant bacteria are not strongly suspected or proven [12]. For patients with an amebic liver abscess, metronidazole (500–750 mg orally TID for 7–10 days) is the drug of choice. Following therapy for invasive amebiasis, treatment with a luminal agent (paromomycin, iodoquinol, or nitazoxanide) to eliminate intraluminal cysts is warranted, even if stool microscopy is negative [3]. Patients are otherwise at risk of relapsing from residual infection in the intestine. In stable patients with a hepatic abscess, antibiotics may be deferred until postaspiration/drainage to increase culture yield [13].

Liver abscess is a rare but serious complication of Crohn's disease with an incidence of 114–297 per 100,000 of patients with Crohn's disease [14]. This is higher than the incidence in the general population (8–16 per 100,000) [14]. Patients with Crohn's disease are at risk for pyogenic liver abscess due to immunosuppressive therapy, fistulous disease, and intraabdominal abscesses [15]. Inflammatory bowel disease patients are also known to have a greater prevalence of amebiasis compared to the rest of the population [16]; however, a higher incidence of amebic liver abscesses has not been reported. Our patient was likely predisposed to such an amebic abscess due to a combination of his travel history and immunosuppression.

## 4. Conclusion

In summary, we describe a case of a liver abscess in a patient with Crohn's disease that was initially presumed pyogenic but later determined to be amebic in origin. Distinguishing amebic from pyogenic liver abscess is important because their treatments and prognoses differ. Epidemiology and serology are two ways to help differentiate the two, as clinical presentations are often similar.
